# Symptoms Removed by Remedies during Treatment of Cases

**Published:** 1891-11

**Authors:** A. L. Kennedy

**Affiliations:** Secretary Boston Hahnemannian Association; Boston


### SYMPTOMS REMOVED BY REMEDIES DURING TREATMENT OF CASES.

BOSTON, July 20th, 1891.

EDITOR OF THE HOMŒOPATHIC PHYSICIAN:

I send herewith for publication a list of symptoms removed by remedies administered during the management of clinical cases by different members of the Boston Hahnemannian Association.

This is the outcome of a method adopted by us in our Association for the purpose of verifying symptoms already found in the pathogeneses of remedies, and also to note any symptoms that are removed by the use of these remedies, but which, as yet, have not appeared in our materia medica.

These symptoms are so arranged as to permit of ready admission to our repertoires. The symptoms marked with a circle (°) are clinical and are not to be found in the provings.

Sincerely yours,

A. L. KENNEDY,

*Secretary Boston Hahnemannian Association.*

### CASE I.

KALI-CARB.$^{cm}$, one dose, dry.

Chest: transient, sharp pains in right side.

Cough: gagging.

Expectoration: rusty; bloody.

Respiration: wheezing.

Sleepiness during day.

Agg.: motion; coughing; breathing; *lying on painful* side; after midnight.

Amel.: lying on painless side.

Remarks: These symptoms occurred in pneumonia.

The patient, though advanced in years, made a good recovery without further medication. *Bryonia* failed to relieve.

The determining difference that led to the choice of *Kali-carb.* in this case lies in the condition of aggravation.

With *Kali-carb.* the patient is *worse* lying on the painful side, while with *Bryonia* he is *better* lying on the painful side.

### Case II.

CALC-C.$^{cm}$, dry, one dose.

Breathing: asthmatic; wheezing.

*Agg.:* motion; pressure of clothes; ° sultry weather; winter; working in cold water.

*Amel.:* autumn; ° north-east rain.

Expectoration viscid.

Asthma twenty-six years' standing.

Remarks: This was a case of asthma of twenty-six years' standing.

A slight aggravation followed the administration of the remedy, and then the case went on to full recovery without further medication.

### Case III.

CARB-VEG.$^{cm}$, dry, three doses.

Vision: illusions of color: ° variegated; ° striped.

° Body feels smaller.

° Walls of room seem falling inward.

Agg.: night; before menses; after menses.

Remarks: This was a case of epilepsy of fifteen years' standing. The attacks were always ushered in with the above symptoms.

No return of the convulsions during fourteen months.

### Case IV.

VERAT-ALB.$^{cm}$, one dose, dry.

Menstruation, before: hot hands and feet.

Menstruation, during: nausea; diarrhœa; cramps in thighs; ° pain in ovarian regions extending down thighs; fainting with pains.

Remarks: These symptoms occurred in a case of dysmenorrhœa that had withstood various forms of treatment for twelve years. The next menstrual period after the remedy was the most agonizing she had ever passed through.

All the symptoms were present except the diarrhœa. Sac-lac. was given.

Four weeks later the next period came, and was normal in every respect.

### Case V.

Baryta-carb.$^{cm}$, one dose, dry.

Scalp: dry, thick, yellow crusts on vertex; falling out of hair; itching, night worse before and during sleep; dryness.

Enlarged cervical glands.

Sweats easily.

Thirst.

"In two weeks the crusts were all off, and the space in the vertex about three inches in diameter was as smooth as a billiard ball; but the hair soon grew out.

"The swelling of the cervical glands, which had been slight, was improved."

### Case VI.

Mag-phos.

Forehead: ° Dull pain.

Vertigo.

Epigastrium: ° dull pain; tenderness to pressure.

Agg.: ascending motion, stooping, lifting. Amel.: rest.

° Menses: late, scanty.

During menses: sharp, transient pains, in hypogastrium; come and go suddenly; ° dull pain in hypogastrium, extending down inside the left thigh to knee; ° bearing-down pains in uterine region.

Agg.: standing.

Amel.: heat, doubling-up pressure, lying down.

"There has been no headache since, and the last two menstruations have been regular and more copious, with but slight discomfort. From being pale, sickly looking, she looks well and has good color."

### Case VII.

Euphrasia$^{cm}$ (Johnstone), one dose.

Eyes: inflamed; photophobia; lachrymation acrid.

Nose: discharge watery, bland, sneezing.

"Had suffered from this condition for twenty-four hours. After the Euphrasia it all disappeared in three hours."

### Case VII.

Sanguinaria$^{dmm}$ (Swan), one dose.

Nose: coryza following soreness in the throat; prickling; sneezing; discharge watery, bland.

"These colds usually lasted two or three weeks. This was all gone in two days."

### Case IX.

Psorinum.

Epigastrium: ° sharp pain extending to back.

° Nausea after eating.

Vomiting, sour mucus.

Urine: scanty, thick, white sediment.

Amel.: ° *hard* pressure.

Agg.: ° one hour after eating; ° pressure of clothing.

These symptoms occurred in a patient whose disease had been diagnosed by a prominent old-school physician as cancer of the stomach.

The patient was rapidly wasting, and had become markedly cachectic. Psorinum was administered for the purpose of arousing the reactive power of the system.

A second appearance of the symptoms was removed by Psorinum.

No further medication was needed.

### Case X.

Natr-mur.

Mind: anxiety, depression, indifference to living; irritable; then indignant because pregnant; ° wants an abortion; predicts death without it; desires to be alone; aversion to husband.

Mouth : sensation of dryness ; saliva increased ; viscid.

Violent thirst : hiccough.

Nausea ; constant, °better by eating.

Vomiting : frothing, stringy, watery, bloody mucus, bile, after eating or drinking.

Stomach : burning, gnawing, better by eating.

° Hunger at four A. M.

Weakness.

Emaciation.

This was a case of a woman twenty-eight years of age—a brunette, and six weeks pregnant. Fourth child. The following remedies were given without avail : Ipec., Colch., Sulph., Sepia, Bry., Mag-mur., Kreos., and Acon.

Natr-mur. removed all the symptoms, including the mental, and the patient went to full term all right.

### CASE XI.

SEPIA.

Cough : paroxysmal ; wakens from sleep ; prevents falling asleep.

Expectoration : greenish.

Agg. : night.

Urination : °delayed, causes flushing and sexual desire ; no desire for.

These symptoms occurred in a woman at the eighth month of pregnancy.

### CASE XII.

ARS.

*Nose bleed after vomiting.*

### CASE XIII.

ARNICA$^{30cm}$.

° Occiput and nape : pain, inclining head backwards.

Nape : grating sensation. Impatience. Vertigo. Nausea, °worse rising in morning.

Rush of blood to head.

° Feet and hands cold.

Pillow seems hard.

Menses early, blood ° dark red, profuse.

*Amel.*: dark room; rest; ° binding up head; cold applications; sitting.

Agg.: light; noise; binding up hair.

This was a case of severe neuralgic headache in a woman of fifty; tall, thin, and dark complexioned.

Attacks occurred at intervals of four to ten days.

$Arnica^{30}$ relieved, and $Arnica^{cm}$ removed all the symptoms, of which there has been no return.

### Case XIV.

$\text{Stannum}^{dmm}$, Swan, one dose.

Hoarseness: with mucus in fauces and larynx.

Agg.: morning; talking; reading aloud.

Amel.: expectoration of mucus.

### Case XV.

Calc-carb.

Blonde, blue eyes.

Mind: disinclination for work.

Urinary organs: ° sensation of weakness in bladder; *urination frequent.*

Agg.: night; in bed; wet weather; alcoholic drinks; coition.

In this case the man had suffered from the urinary symptoms alternating with a headache, for several years, since having an attack of gonorrhœa, for which he was treated by injections.

The symptoms had lasted several months, and were cured within a few days following the remedy.

It is now seventeen months since the symptoms were removed, and there has been no return of the urinary symptoms, the headache, or of the gonorrhœa.

### Case XVI.

Verat-alb.

Mind: irritability; dullness of intellect; forgetfulness.

Chest: ° sharp pains across, after eating.

Stool before: ° bleeding from hæmorrhoids.

Stool during: ° tearing, and severe sharp pain in rectum: ° straining; faintness; cold sweat; nausea.

Stool after: weakness, especially lower extremities.

These hæmorrhoids had existed since before parturition, which occurred three months previously, but latterly had become much more painful.

Relief from Veratrum was prompt, and the cure permanent.

